# Metabolic effects of vasopressin in pathophysiology of diabetic kidney disease

**DOI:** 10.3389/fendo.2023.1176199

**Published:** 2023-09-18

**Authors:** Svetlana Lebedeva, Arus Margaryan, Elena Smolyarchuk, Andrey Nedorubov, Maria Materenchuk, Alexander Tonevitsky, Kerim Mutig

**Affiliations:** ^1^Department of Pharmacology, Institute of Pharmacy, I.M. Sechenov First Moscow State Medical University, Moscow, Russia; ^2^Faculty of Biology and Biotechnology, HSE University, Moscow, Russia; ^3^Department of Translational Physiology, Charité-Universitätsmedizin, Berlin, Germany

**Keywords:** diabetic kidney disease, glucose metabolism, vasopressin, vasopressin V1a receptor (V1aR), vasopressin V1b receptor (V1bR)

## Abstract

The diabetic kidney disease (DKD) is the major cause of the chronic kidney disease (CKD). Enhanced plasma vasopressin (VP) levels have been associated with the pathophysiology of DKD and CKD. Stimulation of VP release in DKD is caused by glucose-dependent reset of the osmostat leading to secondary pathophysiologic effects mediated by distinct VP receptor types. VP is a stress hormone exhibiting the antidiuretic action in the kidney along with broad adaptive effects in other organs. Excessive activation of the vasopressin type 2 (V2) receptor in the kidney leads to glomerular hyperfiltration and nephron loss, whereas stimulation of vasopressin V1a or V1b receptors in the liver, pancreas, and adrenal glands promotes catabolic metabolism for energy mobilization, enhancing glucose production and aggravating DKD. Increasing availability of selective VP receptor antagonists opens new therapeutic windows separating the renal and extra-renal VP effects for the concrete applications. Improved understanding of these paradigms is mandatory for further drug design and translational implementation. The present concise review focuses on metabolic effects of VP affecting DKD pathophysiology.

## Metabolic syndrome and diabetic kidney disease

Metabolic syndrome is a combination of homeostatic deviations associated with a high risk of cardiovascular complications, such as dysregulation of lipid metabolism, high blood glucose levels, and increased blood pressure. Development of the metabolic syndrome is frequently accompanied by insulin resistance, which is the main pathogenetic mechanism of the type 2 diabetes mellitus (DM2) (1, 2). Therefore, a large proportion of people with DM2 displays a complex picture of metabolic pathophysiology encompassing glycemic and non-glycemic components (1, 2). The etiology and pathogenesis of DM2 comprise genetic predisposition, obesity, sleep time deficit or excess, as well as other factors associated with development of insulin resistance and impaired insulin response to glucose or non-glucose stimuli (1, 2). Because of the insulin resistance, DM2 is also referred as insulin-independent DM type. Etiology of the type 1 diabetes mellitus (DM1) includes genetic risk factors triggering pancreatic islet autoimmunity followed by insufficient insulin production and release. Apart from the genetic background, DM1 may be provoked by environmental influences such as intoxications, pancreatic infections, or cancer (1). Independently on the etiology, both DM1 and DM2 lead to progressive reduction of β-cell mass or impaired β-cell function with hyperglycemia as a clinical manifestation. People with hyperglycemia are at risk of major diabetes mellitus complications independently on the diabetes type (1, 2).

Diabetic Kidney Disease (DKD) belongs to frequent and severe complications of both insulin-dependent DM1 and insulin-independent DM2. Clinical features of DKD are largely similar in the two diabetes mellitus types, typically manifesting as enhanced urinary albumin excretion, general proteinuria, reduction of the glomerular filtration rate, disorders of electrolyte and acid-base homeostasis, or hypertension, depending on the disease progression (3). The functional renal deteriorations are strongly related to pathomorphological alterations of kidney tissue encompassing thickening of glomerular and tubular basement membrane, mesangial expansion, glomerulosclerosis, sterile inflammation, arteriolar hyalinosis, and tubulointerstitial fibrosis (4). Even though the initial pathohistological kidney damage patterns differ in patients with DM1 vs. DM2, the resulting glomerular dysfunction and morphological injury due to chronic hyperglycemia appear to play the decisive role in progression of DKD to Chronic Kidney Disease (CKD) with ensuing end stage renal failure (1, 3, 4). Along with hypertension, DKD is the most common etiologic factor of CKD (NIH) (3, 5).

CKD is caused by a heterogeneous group of disorders and characterized by the presence of morphological kidney damage or decline of renal function during three months or longer, irrespective of the cause (6). The severity of CKD is typically assessed by the grade of Glomerular Filtration Rate (GFR) reduction, albuminuria, abnormalities in urinary sediment, as well as morphological kidney damage detected by imaging techniques or histopathological biopsy analysis (6).

Despite diversity of the etiologic factors, the pathogenetic mechanisms of CKD leading to its progress to the end-stage renal failure are intersecting and include Renin-Angiotensin System (RAS) hyperactivity, glomerular hyperfiltration with ensuing glomerusclerosis, renal vasculopathy, as well as cytokine dysregulation and activation of pro-fibrotic pathways (7–9).

In this context, epidemiologic studies revealed an association between increased vasopressin (VP) plasma levels and CKD suggesting a role of excessive VP signaling in pathophysiology of chronic kidney disorders (10). Elevated plasma VP concentrations are typically observed in DM1 and DM2 patients (11–13). Animal models of DM exhibit increased VP levels as well (10, 12). There are several lines of evidence suggesting that exaggerated VP signaling aggravates the course of DKD via renal and systemic effects including dysregulation of glucose and lipid metabolism. VP antagonists have been increasingly recognized as emerging pharmacological strategies for prevention or retardation of CKD of diabetic and non-diabetic origin (10, 14).

While renal physiological functions of VP has been well characterized (15–18), available information on its metabolic effects in other organs and tissues is in part controversial (14). The global trend, however, is suggestive of a pathophysiological impact of VP effects on glucose metabolism in DM2 (12, 14, 19, 20). These pathophysiological effects are mediated by distinct VP receptor types providing a translational perspective of their selective targeting towards corrections of certain metabolic deviations such as hyperglycemia (14).

Apart from DM, experiments in animal suggested that enhanced VP signaling aggravates the fructose-induced metabolic syndrome (21). Improved understanding of metabolic effects and signaling mechanisms involved in the pathogenesis of DM and DKD is mandatory for clinical implementation of selective VP receptor antagonists or agonists. This concise review summarizes recent progress in this direction, with particular focus on the glucose metabolism in DM and DKD.

## Vasopressin and its receptors

The neurohypophysial hormone VP, also referred to as antidiuretic hormone (ADH), fulfils multiple physiological tasks comprising preservation of water homeostasis, regulation of cardiovascular function, stimulation of hormone secretion from anterior pituitary, adjustment of glucose metabolism, and modulation of social behavior (14, 17).

VP is synthesized in the hypothalamus in form of a pre-hormone containing VP, neurophysin, and copeptin (17). After the ensuing cleavage of the pre-hormone in the neurohypophysis, all three components are released into the blood in equimolar amounts. Moreover, plasma copeptin and VP levels closely correlate over the wide range of plasma osmolalities (22). Since detection of VP in plasma is by far less reliable and more complicated than measurement of copeptin, the latter has been established as a surrogate for VP plasma levels in the clinical routine (17, 22, 23).

In addition to the peripheral VP secretion, vasopressinergic neurons of hypothalamus project to other brain regions, where they modulate diverse Central Nervous System (CNS) functions (24). The blood-brain barrier prevents infiltration of VP from the blood into the brain tissue, thus dissociating between its central vs. peripheral actions. The present review work focuses on the peripheral VP effects in the context of DKD and CKD.

VP is a nonapeptide hormone acting via three receptor types: vasopressin V1a (V1aR), V1b (V1bR), and V2 receptors (V2R) (17). The tissue distribution of these receptor types determines the local mode of VP action. V1aR and V1bR show broad expression patterns across the organs and tissues, whereas expression of V2R is limited to the kidney (14).

Despite substantial previous efforts, cell type-specific distribution of distinct VP receptor types is still subject of debates for many organs. The underlying reasons are partially related with a limited availability of selective and robust antibodies to the individual VP receptor types due to a high homology between the VP receptor types and their low expression levels typical for the most G protein-coupled receptor (25).

Therefore, available localization data substantially rely on mRNA techniques and functional studies. Immunolabeling using commercial and non-commercial antibodies produced controversial results (15, 26). Nevertheless, recent development of selective antibodies to V1aR and V2R has enabled their cell type-specific localization in the rodent and human kidneys by knockout tissue-controlled studies (15, 16). Taken together, reliably detectable levels of V1aR expression and significant functional effects of V1aR activation have been reported in the brain, kidney, liver, heart, adrenal glands, and peripheral arteries. V1bR is less broadly distributed among organ and tissues compared to V1aR but is present at least in the pancreas and adrenal gland. Finally, significant V2R expression was detected only in the kidney tissue. The data on organ and tissue distribution of VP receptor types in rodent and human species relevant for the present review are summarized in the **Table 1**.

**Table 1:** Distribution of distinct VP receptor types in mammalian organs and tissues.

Organ/tissue/cell type; (references)	V1a	V1b	V2
*Brain* Hippocampus (27, 28) Arcuate nucleus (27) Solitary tract (27) Inferior olive (27) Brainstem (27) Hypothalamus (27, 28) Amygdala (28) Cerebellum (28) Pituitary gland (14, 27–30)	+ + + + + + - - +	+ + - - - + + + +	- - - - - - - - -
*Kidney* Gl (31) PT TAL (15, 27, 32, 33) MD (15, 16, 34) DCT (15, 27, 32, 33) CNT/CD, PC (15, 27, 32, 33) CNT/CD, IC-A (14, 16, 26, 32, 35) CNT/CD, IC-B (14, 16, 26, 32, 35)	+ - - + (mouse only) - - + +	- - - - - - - -	- - + - + + - -
*Liver* Hepatocytes (27, 32) Cholangiocytes	+ -	- -	- -
*Pancreatic islets* alpha-cells (36, 37) beta-cells (19, 36) delta-cells (36) PP-cells (36)	- + - -	+ + + -	- - - -
*Adrenal gland* Zona glomerulosa (38–40) Zona fasciculate (38–40) Zona reticularis (38–40) Medulla (38–40)	+ + + -	- - - +	- - - -
*Arteries* (14, 16, 27, 31, 32)	+	–	–

**Figure 1:**
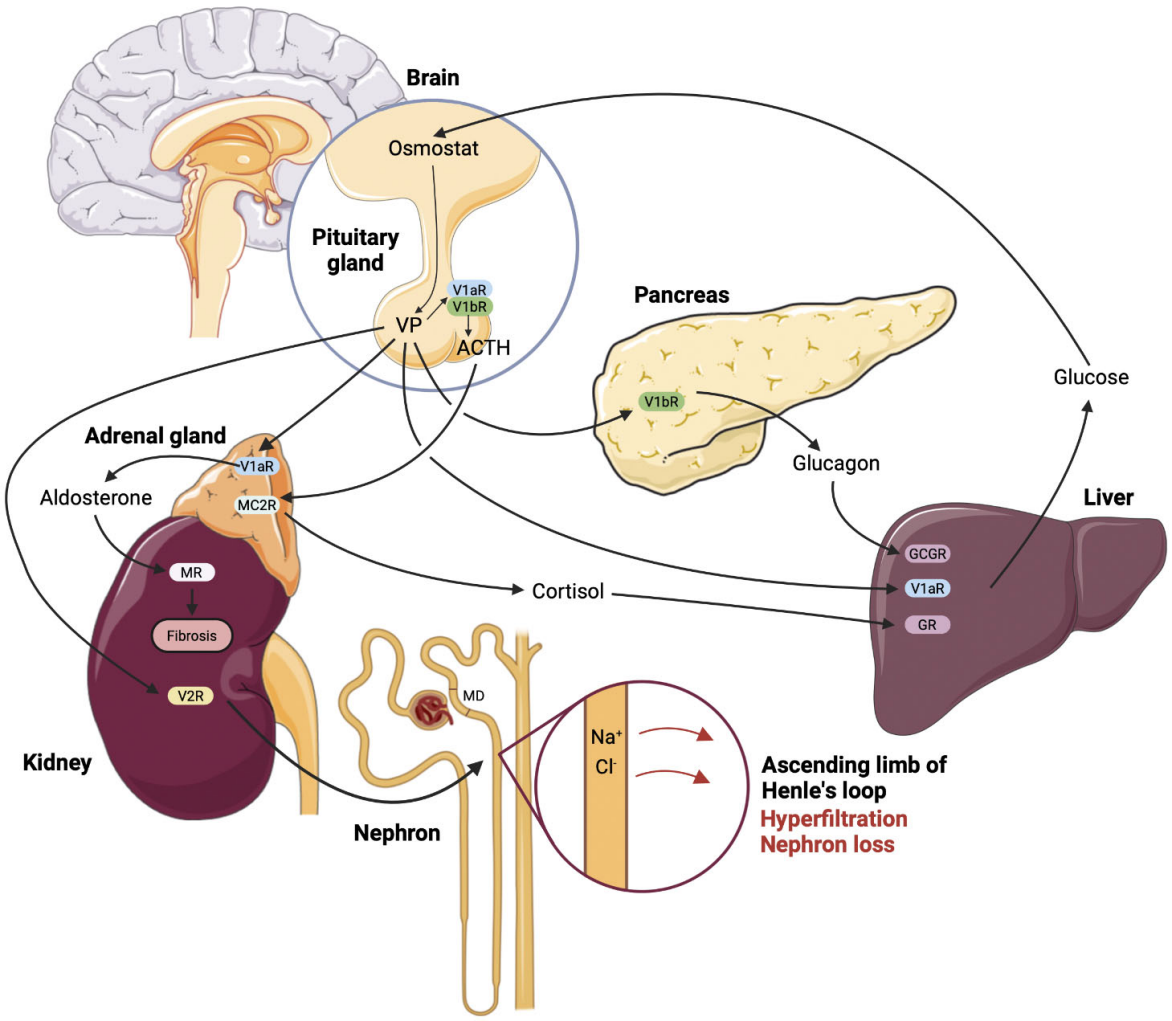
Renal and metabolic nephrotoxic effects of vasopressin mediated by the individual receptor subtypes in diabetic kidney disease. According to the current state of understanding, enhanced plasma glucose levels promote vasopressin (VP) synthesis and release due to reset of the osmostat in hypothalamus. Enhanced circulating VP causes glomerular hyperfiltration with ensuing glomerular damage via sustained stimulation of NaCl reabsorption in the thick ascending limb, decreased NaCl concentration at the macula densa (MD) site and resulting increase of glomerular filtration rate (GFR). This effect is aggravated by renin-angiotensin-aldosterone system hyperactivity caused by VP-induced increase of adrenocorticotropic hormone (ACTH) and aldosterone release, the latter is stimulated directly in the adrenal gland, as well as via the MC2R receptor to ACTH. Parallel release of cortisol potentiates effects of VP on the glucose production in the liver, whereas VP-dependent glucagon release further aggravates the hyperglycemia. Finally, enhanced blood glucose levels maintain high VP plasma levels promoting the renal damage in DKD. The effects of VP are indicated by arrows and the respective receptor type are specified using abbreviations.

The **Table 1** summarized evidence on expression of distinct vasopressin (VP) receptor types across mammalian organs and tissues with focus on their implications in nephrotoxic effects depicted in the **Figure 1**. The data rely on original localization and functional studies, as well as on comprehensive review papers. The presence of the distinct VP receptor types is indicated by (+), whereas the absence of the VP receptors or debatable data are labeled by (-). The respective organ/tissue-specific references are provided in brackets. Gl – glomerulus, PT – proximal tubule, TAL – thick ascending limb, DCT – distal convoluted tubule, CNI – connecting tubule, CD – collecting duct, PC – principal cells, IC – intercalated cells (type A or B).

### V1aR distribution

Early studies of V1aR expression in rat liver and kidney using radioactive *in situ* hybridization revealed presence of V1aR mRNA in hepatocytes, as well as in distal tubular segments of the kidney and renal vasculature (27, 32). The renal finding were largely reproduced in a later autoradiographic V1aR localization using a specific ligand (48). Evaluation of microdissected rat nephron segments and vessels using RT-PCR suggested that V1aR is expressed in renal arteries, along the cortical and medullary CD, as well as in glomeruli (31).

Application of different anti-V1aR antibodies in rodent or human kidney tissues produced controversial results with respect to its segmental and intracellular localization in mammalian kidney (16, 26, 49). Presence of V1aR at the protein level has been convincingly demonstrated in renal vasculature and intercalated cells (IC) of CNT/CD throughout the rodent and human species (16, 35). Mouse but not rat or human kidney exhibited V1aR protein in macula densa (MD) cells as well (16, 34).

Apart from the liver and kidney, expression and functional significance of V1aR have been well established in the cardiovascular system (14, 50, 51). Furthermore, functional studies suggested that V1aR is expressed in the adenohypophysis and adrenal glands with modulating functions in the endocrine homeostasis (29, 38). The V1aR expression in the adrenal gland has been verified in human tissue (39, 40). Therefore, V1aR-mediated stress response may aggravate renal damage in DKD or CKD.

### V1bR distribution

V1bR expression has been mainly localized to various regions of the brain including the anterior pituitary with effects on the adrenocorticotropic hormone (ACTH) release (14, 30). The other reported sites of V1bR expression include pancreas and adrenal glands (28, 36, 40). Overall, long-term V1bR hyperactivity may boost the Renin-Angiotensin-Aldosterone System (RAAS) and cause adverse metabolic effects in DKD patients.

### V2R distribution

V2R receptor is generally considered as the kidney-specific VP receptor type (27, 32, 52). In the kidney, V2R mRNA has been detected along the entire distal nephron, comprising the thick ascending limb (TAL), the distal convoluted tubule (DCT), and the connecting tubule (CNT), as well as in collecting duct (CD) (26, 27, 32, 33). CNT and CD contain two cell types: the principal (PC) and the intercalated cells (IC). Expression of V2R is limited to PCs in these segments (33). In line with the mRNA data, V2R protein has been localized to the basolateral membranes of TAL, DCT, and PCs of CNT/CD (15). The only V2R-negaive cell types within the distal nephron and CD system were the MD cells in the cortical TAL and ICs in CNT/CD (15). The kidney-specific V2R distribution pattern is compatible with the critical role of the VP-V2R signaling in the urinary concentration (53).

## Vasopressin system in diabetic kidney disease

DKD belongs to frequent complications of either type 1 (insulin-dependent) or type 2 (non-insulin-dependent) diabetes mellitus. Along with hypertension, DKD is the most common cause of CKD (3). Diabetic patients usually have elevated plasma VP levels, which may be associated with a resetting of the osmostat or increased fluid turnover (11–13, 54). Chronically enhanced VP secretion may provoke the development or aggravate DKD via renal and extra-renal adverse effects.

## Renal effects of vasopressin in diabetic kidney disease

Experiments in rodent DKD models showed that excessive VP signaling is associated with kidney hypertrophy, glomerular hyperfiltration, and increased albumin excretion (10, 12, 18). Similar symptoms typically occur in diabetic patients prior to the development of DKD (3, 12). Results of chronic infusion of a selective V2R agonist dDAVP (1-deamino-D-arginine8 vasopressin) in normal and VP-deficient Brattleboro rats suggested that the aforementioned deleterious effects of VP are, at least partially, mediated by V2R (18, 55–57). The underlying pathophysiological mechanisms may be related to sustained stimulation of the urinary concentration promoting the NaCl reabsorption along the medullary thick ascending limb (mTAL) followed by inhibition of the tubuloglomerular feedback (TGF) mechanism and compensatory increase in the GFR (18, 57). Chronic increase of GFR is a reasonable mechanism of glomerular hyperfiltration and albuminuria in DKD (12, 57). Nephroprotective effects of V2R antagonists such as tolvaptan has been increasingly recognized, which led to their clinical approval as a part of therapy in patients with Autosomal Dominant Polycystic Kidney Disease (ADPKD) (58). Little evidence is currently available on effects of V2R antagonism in patients with DKD. Retrospective studies point to beneficial effects, as judged by milder histopathological renal damage in kidney biopsies from patients with heart failure and concomitant DKD treated with tolvaptan compared to equivalent patients without tolvaptan administration (59). Apart from that, tolvaptan appears to be instrumental for alleviation of nephrotic syndrome in DKD patients (60). Taken together, V2R antagonists bear therapeutic potential as a part of DKD treatment strategy but their pronounced diuretic action and certain hepatotoxicity may limit patient compliance and complicate the long-term application in chronic kidney disorders such as DKD or CKD (61).

While V2R-activation appears to induce glomerular hyperfiltration via the TGF mechanism, stimulation of V1aR may cause renal vasoconstriction via direct vascular effects (62). In the physiologic situation, the vasoconstrictive effects of VP are buffered by NO and prostaglandin systems (62). Diabetes mellitus impairs the autoregulation of renal blood flow and regulation of GFR via complex pathophysiologic mechanisms affecting K^+^ and Ca^2+^ channel activities in smooth muscle cells and paracrine modulation of the vascular tone (63). The net effect of these changes is pre-glomerular vasodilation and glomerular hyperfiltration with ensuing glomerular damage leading to glomerulosclerosis (3, 63). Apart from vascular effects, V1aR mediates stimulation of H^+^ secretion by type A ICs (IC-A) in response to VP, thereby promoting urinary acidification (16). This effect may be related to potentiation of aldosterone action upon concomitant V1aR activation in ICs (35). Whether V1aR-dependent modulation of urinary acidification plays a role in pathophysiology of DKD remains to be clarified. Finally, VP binding to V1aR in MD cells has been shown to increase renin secretion with resulting systemic RAS activation (16, 34). Presence of V1aR in MD cells was demonstrated in the mouse species only, whereas localization of the receptor in rat and human kidney failed to confirm this result (16). Despite potential interspecies differences in V1aR-dependent regulation of renin release, extra-renal stimulating effects of VP on RAAS activity such as enhanced ACTH or adrenal hormone secretion were extensively documented in rodent and human species (28, 29, 38, 40, 41, 64).

## Effects of vasopressin on renin-angiotensin-aldosterone system in diabetic kidney disease

Several clinical studies reported increased RAAS activity in patients with type 2 diabetes, especially in the presence of DKD (65–68). RAAS hyperactivity has been well recognized as a critical pathogenetic factor contributing to progression of DKD and CKD towards advanced renal fibrosis and end-stage kidney disease (3). There is growing clinical and scientific evidence suggesting that effects of VP in different organs synergistically stimulate RAAS. VP modulates the neurohypophyseal-adrenal axis by promoting the ACTH release in the neurohypophysis, increasing the sensitivity of adrenal glands to ACTH, and enhancing the adrenal synthesis and secretion of steroid hormones including aldosterone (28, 38, 40, 69, 70). These effects are mediated by V1aR or V1bR. VP-deficient Brattleboro rats showed a dissociation between high plasma renin and low plasma aldosterone levels along with decreased amount of angiotensin II (AngII) binding sites in the adrenal glands reflecting the idea that VP may potentiate effects of AngII via regulation of the AngII receptor type 1 (AT1R) (71). Moreover, VP and AngII have been shown to strengthen effects of each other in the kidney, which may be related to the shared downstream cAMP-dependent signaling pathways (71, 72).

In contrast to hyperreninemia observed in Brattleboro rats with global suppression of VP signaling, selective deletion of V1aR in mice was associated with reduced renin expression and plasma activity (34). This discrepancy may be explained by interspecies differences in renal V1aR expression in MD cells and VP-dependent paracrine stimulation of renin-producing juxtaglomerular cells (34). Unlike mouse species, MD cells in rat and human kidney are devoid of V1aR (16). VP has been shown to suppress renin expression and plasma activity in rat and human species via the V2R-mediated signaling (44, 71, 73).

Taken together, elevated VP levels in DKD may increase the RAAS activity at different levels including stimulation of ACTH and aldosterone release, modulation of adrenal sensitivity to AngII, and direct synergism with AngII via intracellular cAMP generation. These effects are mediated by V1aR or V1bR. In contract, activation of V2R may counterbalance the RAAS hyperactivity via suppression of renal renin expression and release. The net effect of all three VP receptor types on distinct RAAS components may be variable but clinical reports are suggestive of RAAS activation in DM patients (65, 68). In particular, enhanced aldosterone plasma levels have been closely associated with insulin resistance and DKD (68, 74). In the classic paradigm, aldosterone is considered as a Na^+^-sparing and K^+^-secreting hormone predominantly acting via modulating the relevant gene expression in the aldosterone-sensitive distal nephron (ASDN) of the kidney, which comprises the late distal convoluted tubule (DCT2), connecting tubule (CNT), and cortical collecting duct (cCD) (75). However, recent progress in understanding of genomic and non-genomic effects of aldosterone mediated by the mineralocorticoid receptors (MR) or alternative vascular aldosterone-sensitive pathways has broadened the interpretation of its role in induction and aggravation of CKD and renal fibrosis (76). According to the accumulated clinical and scientific data, effects of aldosterone on the kidney function and morphology are wider than just regulation of electrolyte handling in CNT and CD. Aggravating effects of elevated plasma aldosterone levels in DKD may be direct or indirect including the well-known renal axis and recently identified alternative pathways. In this context, suppression of aldosterone release using V1aR or V1bR antagonists may contribute to the renoprotection in patients with DM.

## Metabolic effects of vasopressin in diabetic kidney disease

### Effects of vasopressin on glucose metabolism

As a stress hormone, VP exerts numerous direct and indirect metabolic effects towards energy mobilization. Glucose is a principal energy source for the brain and an important secondary energy substrate for many other organs and tissues. Importantly, the process of urine concentration requires glucose as the energy source since the medullary portions of the distal nephron and collecting duct system function upon the physiologic hypoxia and utilize glucose. Moreover, adaptations of glucose metabolism in renal epithelial cells has been implicated in pathophysiology of acute and chronic renal injuries (77). VP regulates the glucose metabolism via central and peripheral effects resulting in elevation of blood glucose levels (78). Direct effects of VP on glucose availability in the blood are mediated by V1aR in the liver and V1bR in the pancreas (27, 32, 36). Binding of VP to V1aR in the liver induces glycogen degradation thereby augmenting the blood glucose levels (32). At the same time, activation of V1bR in alpha cells of pancreatic islets stimulates glucagon release, which further promotes glycogenolysis in the liver (36). VP has been shown to increase plasma insulin levels as well, although to a lesser extent compared to glucagon (78). Since V1bR expression was predominantly reported in the glucagon-producing alpha cells of pancreatic islets, effects of VP on the insulin release are likely indirect and may be mediated by enhanced glucose concentrations in the blood (79). Stimulation of cortisol release in response to VP may further contribute to rise in blood glucose levels by promoting gluconeogenesis in the liver (41, 42). Apart from direct and indirect stimulation of the glucose production, VP has been shown to modulate insulin sensitivity and lipolytic activity in the adipose tissue, which may secondarily affect the blood glucose concentration as well (80). The net effect of the complex physiological action of VP on the glucose metabolism is an increase of the blood glucose concentration likely serving to promote the global adaptation of the energy metabolism to stress.

Enhanced VP signaling has been increasingly recognized as a critical factor aggravating the metabolic syndrome in DM2 and promoting the kidney damage in DKD and CKD (18, 57). Effects of VP on the glucose production are distinctly mediated by V1aR or V1bR, enabling their selective targeting using receptor-specific agonists or antagonists. Development of such strategies requires improved molecular understanding of VP signaling downstream of V1aR vs. V1bR. In this context, characterization of transgenic mice with selective deletion of V1aR (V1aR^-/-^) or V1bR (V1bR^-/-^) produced a complex picture with respect to their individual roles in the glucose metabolism (20). Notably, effects of pharmacologic vs. genetic suppression of V1aR and V1bR types produced in part conflicting results demanding for mechanistic explanations (14).

### Effects of pharmacologic vs. genetic V1a receptor inactivation on glucose homeostasis

Studies using acute and chronic administration of V1aR agonists and antagonists in rodents suggested that V1aR activation increases the blood glucose levels but reduces the glucose tolerance (37). These effects are likely mediated by increased production of glucose in the liver and modulation of insulin sensitivity in peripheral tissues. Analysis of V1aR^-/-^ mice showed impaired glucose tolerance as well, but hepatic production of glucose was increased (20, 81). Based on pharmacologic effects of V1aR antagonists, V1aR deletion was expected to suppress glycogenolysis and lower the blood glucose levels. Surprisingly, glycogen content was decreased in the liver tissue of V1aR^-/-^ mice (81). Pharmacologic stimulation of V1aR produced pro-diabetic effects, whereas V1aR antagonism revealed antidiabetic potential in rats (37). In contrast, challenging of V1aR^-/-^ mice with high-fat diet produced more pronounced obesity, hyperleptinemia, and impaired glucose tolerance compared to control mice suggesting that V1aR deletion induces a pre-diabetic condition and aggravates the metabolic syndrome in this model (20, 81). The reasons for discrepant effects of pharmacologic vs. genetic suppression of the V1aR signaling on the glucose homeostasis are still poorly understood but may be related with induction of compensatory mechanisms during embryonal and postnatal development of V1aR^-/-^ mice. Such mechanisms may include enhanced hepatic response to other stimuli for glucose production or *de novo* expression of V1bR in the liver of V1aR^-/-^ mice (21). While V1aR-deficient mice represent a valuable model for improved understanding of VP biology, detailed characterization of effects elicited by selective V1aR agonists and antagonists in rodent models of DM2 and DKD is of immediate clinical relevance (37).

### Effects of pharmacologic vs. genetic V1b receptor inactivation on glucose homeostasis

V1bR is expressed in alpha cells of pancreatic islet and mediates VP-induced stimulation of glucagon release via the Ca^2+^/inositol 1,4,5-triphosphate signaling pathway (19, 36, 78). Glucagon, in turn, increases systemic glucose availability via activation of glycogenolysis in the liver and increasing gluconeogenesis in the liver and kidney. Systemic availability of glucose may be further increased by lipolysis (43). Parallel V1bR-mediated stimulation of catecholamine release may further contribute to hyperglycemic effects of VP (82). The resulting rise in blood glucose concentration may cause a modest increase in insulin plasma concentration, which has been documented in response to VP in several *in vivo* and *ex vivo* studies (79). The idea that effects of VP on the insulin release are indirect is supported by the absence of convincing reports documenting expression of VP receptors in the beta cells of pancreatic islet, as well as by the blunted VP effects on the insulin release in the absence of concomitant increase in the blood glucose concentration. In line with this, effects of VP on the insulin plasma levels were completely abolished in V1bR^-/-^ mice (79). Studies using AVP together with selective antagonists for V1aR, V1bR or a combined V1aR and V1bR antagonist in isolated rodent pancreatic tissue also confirmed that the direct effect of AVP on the glucagon release is mediated by V1bR, whereas the effect on insulin release is indirect (79). Analysis of the glucose homeostasis in V1bR^-/-^ mice revealed decreased fasting glucagon, insulin, and glucose levels in the blood along with increased insulin sensitivity in peripheral tissues (83). Therefore, the data accumulated so far suggests that V1bR mediates effects of AVP on the blood glucose levels and may significantly contribute to the progression of DM2 and DKD.

### Effects of double V1aR and V1bR inactivation and global AVP deficiency on glucose homeostasis

Since both V1aR and V1bR are implicated in the glucose homeostasis, effects of their concomitant deletion were evaluated. The phenotype of the double-knockout mice was largely reflecting the phenotype of V1aR^-/-^ mice and showed higher blood glucose and insulin levels along with impaired glucose tolerance (14, 20). Based on these results it is tempting to speculate that stimulation of V1aR may lower the blood glucose concentration. However, pharmacologic V1aR activation induced hyperglycemia, whereas administration of a V1aR antagonist blunted the AVP-induced hyperglycemia in normal rats (37). In addition, evaluation of the glucose homeostasis in VP-deficient Brattleboro rats revealed lower plasma glucose and insulin levels along with enhanced glucose tolerance (84). The in part conflicting results obtained in different models of pharmacologic or genetic inhibition of VP signaling require improved understanding and cautious interpretation with respect to potential clinical application of V1aR- or V1bR-antagonists in diabetic patients. **Figure 1** provides a concept of VP-induced kidney damage combining renal and metabolic effects.

### Effects of vasopressin on lipid metabolism

VP enables water conservation and at the same time functions as a stress hormone. Fat is a source of metabolic water and an energy source as well. VP exerts central and peripheral modulating effects on lipid metabolism. Indeed, the net effect of VP on the lipid homeostasis depends on the global metabolic status and combines distinct actions in different tissues (14). As a stress hormone, AVP enhances the sympathetic tone thereby promoting lipolysis in the adipose tissue (44–46). Concomitant VP-induced stimulation of adrenal hormone release may have multiple effects on lipid metabolism depending on the current nutrition status (85). In fact, both lipolytic and antilipolytic actions have been reported in rats depending on the experimental conditions (86). In addition to the indirect effects mediated by changes in the vegetative and endocrine status, VP affects the lipid metabolism directly by targeting several organs and tissues. Acting via V1 receptor types, VP has been reported to promote triacylglycerol mobilization in the heart, which may serve to support cardiac energy metabolism (87). However, chronically exaggerated VP signaling has been associated with cardiovascular disorders and combined V1aR/V2R antagonism demonstrated beneficial effects in patients with acute heart failure (50, 51, 88). Activation of V1aR or V1bR in the pancreas and the associated glucagon release may promote hepatic lipolysis via Ca^2+^/inositol triphosphate-dependent signaling in order to enhance the glucose production (43, 47). Peripheral effects of VP in the fat tissue are debatable since both lipolytic and anti-lipolytic actions were reported and distinct underlying mechanisms including direct and hemodynamic effects discussed (80, 86, 89). Analysis of V1aR^-/-^ and V1bR^-/-^ mouse strains suggested that many effects of VP on lipid metabolism may be mediated by changes in insulin sensitivity (90, 91). Taken together, VP exerts complex effects on lipid homeostasis requiring further characterization. The physiological sense of these effects appears to be related to catabolic metabolism and energy substrate supply at short term (14). Chronic increases of circulating VP levels in DM2 may promote the metabolic syndrome and aggravate DKD (**Table 2**).

**Table 2:** Metabolic effects of vasopressin (VP) in diabetic kidney disease.

Direct and indirect metabolic effects	Involved VP receptor type/organ or tissue	Pathophysiologic effects in DKD	References
Glucose metabolism			
Glycogenolysis	V1a/liver	Hyperglycemia	(27, 32)
Glucagone release -> glycogenolysis	V1b/pancreas -> liver	Hyperglycemia	(36)
Cortisol release -> gluconeogenesis	V1a/adrenal glands -> liver	Hyperglycemia	(41–43)
Fat metabolism			
↑ sympathetic tone	V1b or V1a/hypothalamus, pituitary gland, adrenal gland -> fat tissue	Lipolysis -> insulin resistance	(44–46)
Insulin resistance	V1a or V1b/pancreas	Lipolysis -> hyperglycemia	(43, 47)

The **Table 2** summarizes potential adverse metabolic effects of vasopressin (VP) in Diabetic Kidney Disease (DKD). (->) indicates sequence of events, (↑) means an increase.

## Conclusions and perspectives

Apart from the antidiuretic function, VP has been increasingly recognized as a global player in the glucose, lipid, and protein homeostasis. Unlike the V2R-dependent antidiuretic functions, metabolic effects of VP are predominantly mediated by V1aR and V1bR. Excessive V2R-mediated signaling substantially aggravates kidney damage in DKD and CKD. Selective V2R antagonists are available and even approved for treatment of hyponatremia and polycystic kidney disease (92, 93). Despite encouraging perspective of their use in DKD and CKD patients, a relatively high hepatotoxicity and poor patient compliance due to the pronounced diuretic action limit practical implementation of this strategy (61). Pharmacological targeting of V1aR or V1bR may provide metabolic benefits on glucose homeostasis indirectly protecting the kidney. Dysregulation of glucose homeostasis is a part of metabolic syndrome and may be particular relevant in pathophysiology of DM and DKD. Numerous transgenic and pharmacological models provide perspectives for selective V1aR or V1bR modulation. However, these models produced in part conflicting results complicating the clear conclusions. Nevertheless, integrative analysis of the available data suggests that selective antagonists of V1aR or V1bR bear blood glucose lowering potential. Moreover, such antagonists may produce further renoprotective benefits by suppressing RAAS and adrenal stress hormone levels. Clinical experience with V1aR or V1bR antagonists are largely limited to dual V1aR/V2R antagonism in patients with acute heart failure (94, 95). Experiments in rats suggest renoprotective effects of dual V1aR/V2R suppression due to RAAS inhibition (96). Further characterization of V1aR- vs. V1bR antagonists with respect to their metabolic effects may unravel their therapeutic potential in DKD and CKD.

## Author contributions

SL – statement and development of concept, key goals and objectives. Text preparation and editing – critical revision of the manuscript draft with the valuable intellectual investment. AM, AN, ES – text preparation and editing. MM – figure and table preparation, text preparation and editing. KM – statement and development of concept, key goals and objectives, text preparation and editing, approval of the final manuscript – acceptance of responsibility for all types of the work, integrity of all parts of the paper and its final version. AT – discussion of glucose metabolism and critical revision of the manuscript draft with the valuable intellectual investment. All authors contributed to the article and approved the submitted version.
